# Effects of statins on kidney function in older adults

**DOI:** 10.1111/jgs.19319

**Published:** 2024-12-18

**Authors:** Michelle A. Fravel, Michael E. Ernst, Robyn L. Woods, Suzanne G. Orchard, Kevan R. Polkinghorne, Rory Wolfe, James B. Wetmore, Mark R. Nelson, Elisa Bongetti, Anne M. Murray, Sophia Zoungas, Zhen Zhou

**Affiliations:** ^1^ Department of Pharmacy Practice and Science, College of Pharmacy The University of Iowa Iowa City Iowa USA; ^2^ Department of Family Medicine, Carver College of Medicine The University of Iowa Iowa City Iowa USA; ^3^ School of Public Health and Preventive Medicine Monash University Melbourne Victoria Australia; ^4^ Department of Nephrology, Monash Medical Centre Monash Health Melbourne Victoria Australia; ^5^ Department of Medicine Monash University Melbourne Victoria Australia; ^6^ Department of Medicine Hennepin Healthcare Minneapolis Minnesota USA; ^7^ Nephrology Department Hennepin Healthcare Minneapolis Minnesota USA; ^8^ Hennepin Healthcare Research Institute Minneapolis Minnesota USA; ^9^ Menzies Institute for Medical Research University of Tasmania Hobart Tasmania Australia; ^10^ Berman Center for Outcomes and Clinical Research Hennepin‐Health Research Institute Minneapolis Minnesota USA; ^11^ Division of Geriatrics, Department of Medicine Hennepin Healthcare Minneapolis Minnesota USA

**Keywords:** ACR, eGFR, older adult, statins

## Abstract

**Background:**

The effect of statin therapy on kidney function among older adults is unclear.

**Objectives:**

To examine the association between statin use and changes in estimated glomerular filtration rate (eGFR) and urine albumin‐to‐creatinine ratio (UACR), positive or negative, in an older adult cohort with versus without chronic kidney disease (CKD) at baseline.

**Methods:**

This analysis included 18,056 participants aged ≥65 years with versus without CKD at baseline in a randomized trial of low‐dose aspirin, who had no prior cardiovascular events, major physical disability, or dementia initially. Outcome measures included eGFR and UACR. Linear mixed‐effects models were used to estimate the associations of baseline statin use versus no use with eGFR and UACR changes over time. The inverse‐probability of treatment‐weighting technique was used for all analyses to address confounding by indication due to the lack of randomization in treatment assignment.

**Results:**

Statin use was not associated with change in eGFR, UACR, or incident CKD in participants with or without CKD at baseline (*p* > 0.05 for all associations). Subgroup analyses found no significant interactions between statin and age, sex, diabetes, country, and frailty status on any of the study outcomes.

**Conclusions:**

Among adults ≥65 years of age, with and without CKD, statin therapy was not associated with improved or worsened kidney function. This data suggests that the decision to use versus not use statins in this population may be ideally guided by factors other than kidney health.

**Key points**

- In a cohort of 18,056 community‐dwelling older adults from Australia and the United States, use of statin therapy was not associated with improved or worsened estimated glomerular filtration rate and urine albumin‐to‐creatine ratio over time.
- The lack of association between statin use and estimated glomerular filtration rate and urine albumin‐to‐creatine ratio was demonstrated for participants with and without chronic kidney disease (CKD).

**Why does this paper matter?**

This article demonstrates that statin use is not associated with changes in kidney function over time in older adults with or without chronic kidney disease (CKD). While our findings did not uncover a relationship between statin use and improved kidney health, the data supports the kidney safety of statins in this population. These findings do not support the use of statins for preservation of kidney function; however, they do suggest that the decision to use a statin for other indications should not be limited by concerns related to potential kidney harm.

## INTRODUCTION

There is an increasing appreciation for the interconnectedness of cardiovascular disease (CVD) and chronic kidney disease (CKD), as outlined in the 2023 American Heart Association presidential advisory on cardiovascular kidney metabolic health.^1^ The interwoven pathophysiology of cardiovascular and kidney disease is embodied in their reciprocal relationship, where each condition can exacerbate the other.^2^ Metabolic derangements, including abdominal obesity, impaired glucose tolerance, dyslipidemia, and hypertension, underpin the development of both CVD and CKD, leading to the recent recognition and labeling of this constellation of disorders as Cardiovascular‐Kidney‐Metabolic (CKM) Syndrome.^2^

Although the primary role of statin therapy is to lower LDL, the “pleiotropic” effects of statins are well established.^3^ The discovery of these cholesterol‐independent effects, including atherosclerotic plaque stabilization, improved endothelial function, decreased inflammation, antioxidant effects, and increased nitric oxide production, among others, has led to expanded use of statins over the years, given demonstrated benefit in improving cardiovascular outcomes when used in at‐risk patients with “normal” LDL levels.^4^ Mechanisms proposed to explain statin‐induced improvements in kidney function specifically include reduced kidney damage as a result of decreased circulating LDL, improved renal perfusion due to enhanced endothelial function, and enhanced availability of nitric oxide to assist in regulation of renal hemodynamics.^5^

Meta‐analyses of randomized controlled trials including participants with and without CKD at baseline have found an association between statin use and a slower rate of glomerular filtration rate (GFR) decline,^6^, ^7^ while retrospective cohort studies in both populations have reported mixed results.^8^, ^9^ A landmark randomized controlled trial of >9000 adults with CKD (mean eGFR 27 mL/min per 1.73 m^2^, mean age 61 years) found no benefit on the rate of change in estimated glomerular filtration rate (eGFR) with use of simvastatin 20 mg plus ezetimibe 10 mg daily.^5^ While the randomized, prospective design of this study is a notable strength, limitations included lack of participants without CKD at baseline, inclusion of a significant proportion of participants with end‐stage kidney disease (ESKD) receiving dialysis, lower mean age (63 years), and follow‐up limited to 5 years. Notably, instances of kidney harm associated with statin use have also been reported. Rosuvastatin, for example, has been associated with increased risk of hematuria, proteinuria, and progression to ESKD compared to other statins, attributable to renal tubular toxicity, especially when used in patients with CKD treated with high doses.^10^ This has led to an FDA recommendation to limit the dose of rosuvastatin to a maximum of 10 mg daily in patients with CrCL < 30 mL/min.

In contrast to younger populations, in older adults, the decision to use a statin for primary prevention of CVD is complicated by an unclear benefit‐to‐risk ratio. While the absolute risk of a cardiovascular event increases with age, there is a lack of prospective randomized controlled trials demonstrating the safety and efficacy of statins in mitigating CV outcomes among this population. Current guidelines do not provide specific recommendations for use of statins for primary CVD prevention in adults over 75 years; therefore, the decision relies heavily on a clinician‐patient discussion. In appreciation of this point, two large, randomized trials (STAREE, PREVENTABLE) are in progress.^11^, ^12^ If older adults can benefit from statin use in both kidney and cardiovascular health, the value of treatment may outweigh risks. If, on the other hand, statin use is associated with kidney harm among this population, treatment to prevent CVD may be less desirable. To investigate the relationship between statins and kidney health, we examined the association between statin use and rate of eGFR decline and change in albuminuria, in older adults with and without CKD, enrolled in the ASPirin in Reducing Events in the Elderly (ASPREE) trial.

## METHODS

### Data source and population

We conducted a post hoc analysis of data from the ASPREE trial, a randomized, placebo‐controlled trial of daily, low‐dose (100‐mg) aspirin in participants from Australia and the United States from 2010 to 2017 and its long‐term observational follow‐up (ASPREE‐XT, 2018–2022). Details related to the study design and main outcomes have been previously published.^13^, ^14^, ^15^, ^16^ Briefly, ASPREE enrolled 19,114 community‐dwelling adults aged 70 years and older (65 years for US minorities) without CVD, dementia, independence‐limiting physical disability, or chronic illness expected to limit survival to <5 years. All participants provided written informed consent to participate, and the trial was conducted in accordance with the principles of the Declaration of Helsinki and approved by local institutional review boards at each site.

Participants underwent annual study visits which included collection of standardized assessments of physical and cognitive function, anthropometrics, and updates of the medical history and prescription medications. A basic set of laboratory tests were also collected including serum creatinine and urine albumin‐to‐creatinine ratio (UACR) which were obtained at baseline and at every annual visit throughout the study. eGFR was calculated using the 2021 Chronic Kidney Disease Epidemiology Collaboration CKD‐EPI.^17^ Information regarding medication use was gathered from primary care physician records or through self‐report of current medication use during the baseline and all annual visits.

### Study endpoints

Utilizing the KDIGO definition of CKD, which incorporates both eGFR and, separately, UACR thresholds,^18^ CKD was designated by the presence of an eGFR <60 mL/min/1.73 m^2^ and/or UACR > 3 mg/mmol at baseline. We examined the change in eGFR and UACR over time among participants with versus without CKD at baseline in: (1) baseline statin users compared to non‐statin users; and (2) between users of rosuvastatin compared to users of other statins, given specific reports of increased risk of kidney harm associated with rosuvastatin.

### Statistical analysis

For all comparisons, we used the inverse‐probability of treatment weighting (IPTW) technique to address confounding by indication due to the lack of randomization in treatment assignment. The IPTW is an approach based upon propensity scores, which refer to the probability of receiving a statin (or a specific statin class), conditional on observed baseline covariates. We computed propensity score for the use of statins (or a specific statin class) for each participant using multivariable logistic regression models; the dependent variable was baseline exposure to statin (statin class) and independent variables were those associated with statin use and CKD outcomes according to clinical expertise and previous literature. These included age, sex, country (Australia/United States), ethnicity/race (White; Black; Hispanic/Latino/Other), smoking status, alcohol use, education level (<12 years; ≥12 years), mean systolic blood pressure (SBP), mean diastolic blood pressure (DBP), mean body mass index (BMI), hypertension (blood pressure ≥ 140/90 mmHg or on antihypertensive medications) and its treatment (grouped into the following categories: no hypertension; hypertension not on medication; hypertension on medications [no diuretics and no ACEi/ARB]; hypertension on medications [including diuretics but not ACEi/ARB]; hypertension on medications [including diuretics and ACEi/ARB]), diabetes (self‐report or fasting glucose ≥126 mg/dL or on glucose‐lower medications) and its treatment (grouped into the following categories: none; diabetes‐not treated with medications; diabetes‐treated [oral hypoglycemic but no insulin]; diabetes‐treated [on insulin]), polypharmacy, use of nonsteroidal anti‐inflammatory medications, use of proton pump inhibitors, other lipid‐lowering medication use, frailty status, randomized aspirin, baseline eGFR and UACR levels. We then calculated stabilized weights using propensity scores to reweight the study population and truncated weights at the 1st and 99th percentiles. Last, we checked the covariate balance using absolute standardized difference (ASD); an ASD < 0.1 indicates a good balance. The covariates were well‐balanced in all weighted populations (data not shown).

In the study population, the mean values of eGFR and UACR at baseline and each following visit by baseline CKD status were plotted to visualize the kidney function change over time. In the weighted population, we used linear mixed‐effects models to estimate the associations of baseline statin use versus no use with eGFR and UACR changes over time. Both eGFR and UACR were modeled as a continuous variable and we logarithmically transformed UACR due to a skewed distribution. The model encompasses statin exposure, a continuous variable to indicate the annual visit when eGFR or UACR was measured, and a statin‐by‐visit interaction, as well as participant‐specific intercept and slope as random effects. For incident CKD events, participants were followed from the trial commencement to the diagnosis of CKD, death, or the last follow‐up visit with measurement of eGFR and/or UACR, whichever occurred first.

We used IPTW‐adjusted Cox proportional‐hazards regression model to estimate hazard ratios (HRs) for the outcome with baseline statin use in non‐CKD participants. Proportional hazard assumption was checked by Schoenfeld residuals to assure no violation of assumptions occurred. The analysis was repeated with IPTW‐adjusted Fine–Gray subdistribution hazard model to consider competing death events. We also repeated the main analyses in CKD participants and non‐CKD participants in subgroups stratified by age, sex, diabetes, country, and frailty status (pre‐frailty and frailty were combined into one group due to the small sample size of frail participants).

All statistical tests were 2‐sided, and a *p*‐value <0.05 was considered statistically significant. Analyses were performed using Stata SE 17.0 (StataCorp, College Station, TX: StataCorp LLC).

## RESULTS

Beginning with the full cohort of 19,114 ASPREE participants, 970 were excluded due to missing eGFR and UACR data from either baseline or follow‐up and 88 were excluded due to missing covariate data. Of the remaining 18,056 participants included in this analysis, 3802 (21.1%) had CKD at baseline and 14,254 (78.9%) did not (Figure S1).

Table 1 shows the baseline characteristics of participants according to CKD status and statin use. Among the cohort of 14,254 participants without CKD at baseline, the median age was 73.6 years (interquartile range 71.5 to 77.0). Those taking a statin at baseline were less likely to consume alcohol, or have 12 years or more of education, and they were more likely to be female, have a higher BMI, a higher UACR, a lower eGFR, were more likely to be taking a PPI or another lipid‐lowering agent, and have polypharmacy, hypertension, and diabetes, and to be frail compared to those not taking a statin at baseline. In the 3802 participants with CKD at baseline, the median age was 75.7 years (interquartile range 72.5 to 80.2). Those with CKD who were taking a statin at baseline were more likely to be female, have a higher BMI, and were more likely to have a lower eGFR, be taking a PPI or another lipid agent, have polypharmacy, hypertension, and diabetes compared to those not taking a statin at baseline. Additional information regarding statin use among the cohort is provided in Table S1. More participants with CKD at baseline were taking a statin compared to those without CKD (36.7% vs. 30.0%). Among all participants, irrespective of CKD status, atorvastatin was the most commonly used statin (38%), followed by simvastatin (29%) and rosuvastatin (26%).

**TABLE 1:** Baseline characteristics of chronic kidney disease (CKD) and non‐CKD participants in total and by baseline statin use.

Characteristics	Baseline CKD participants				Baseline non‐CKD participants			
	Total (*n* = 3802)	No statin (*n* = 2407, 63.3%)	Statin (*n* = 1395, 36.7%)	*p* value	Total (*n* = 14,254)	No statin (*n* = 9976, 70.0%)	Statin (*n* = 4278, 30.0%)	*p* value
Demographics								
Age, median (IQR), years	75.7 (72.5–80.2)	76.0 (72.6–80.6)	75.2 (72.3–79.5)	<0.001	73.6 (71.5–77.0)	73.6 (71.5–77.1)	73.7 (71.5–76.9)	0.67
Female, *n* (%)	2187 (57.5)	1321 (54.9)	866 (62.1)	<0.001	7999 (56.1)	5402 (54.1)	2597 (60.7)	<0.001
Country, *n* (%)								
Australia	3271 (86.0)	2109 (87.6)	1162 (83.3)	<0.001	12,744 (89.4)	8953 (89.7)	3791 (88.6)	0.045
United States	531 (14.0)	298 (12.4)	233 (16.7)		1510 (10.6)	1023 (10.3)	487 (11.4)	
Ethnicity/Race, *n* (%)								
White	3425 (90.1)	2207 (91.7)	1218 (87.3)	<0.001	13,248 (92.9)	9333 (93.6)	3915 (91.5)	<0.001
Black	236 (6.2)	128 (5.3)	108 (7.7)		494 (3.5)	331 (3.3)	163 (3.8)	
Hispanic/Latino	82 (2.2)	46 (1.9)	36 (2.6)		314 (2.2)	189 (1.9)	125 (2.9)	
Other	59 (1.6)	26 (1.1)	33 (2.4)		198 (1.4)	123 (1.2)	75 (1.8)	
Health behaviors and social determinants								
Smoking, *n* (%)								
Never	2101 (55.3)	1340 (55.7)	761 (54.6)	0.71	7935 (55.7)	5596 (56.1)	2339 (54.7)	0.28
Former	1558 (41.0)	980 (40.7)	578 (41.4)		5809 (40.8)	4030 (40.4)	1779 (41.6)	
Current	143 (3.8)	87 (3.6)	56 (4.0)		510 (3.6)	350 (3.5)	160 (3.7)	
Alcohol use, *n* (%)								
Never	794 (20.9)	477 (19.8)	317 (22.7)	0.054	2294 (16.1)	1558 (15.6)	736 (17.2)	0.02
Former	250 (6.6)	152 (6.3)	98 (7.0)		784 (5.5)	532 (5.3)	252 (5.9)	
Current	2578 (72.5)	1778 (73.9)	980 (70.3)		11,176 (78.4)	7886 (79.0)	3290 (76.9)	
Education ≥ 12 years, *n* (%)	1985 (52.2)	1270 (52.8)	715 (51.3)	0.37	7862 (55.2)	5660 (56.7)	2202 (51.5)	<0.001
Clinical and laboratory measures								
SBP, mm Hg, mean (SD)	141.4 (17.1)	141.8 (17.2)	140.6 (16.8)	0.03	138.6 (16.3)	138.7 (16.4)	138.5 (15.9)	0.49
DBP, mm Hg, mean (SD)	77.1 (10.4)	77.6 (10.5)	76.3 (10.3)	<0.001	77.3 (9.8)	77.5 (9.9)	76.8 (9.7)	<0.001
BMI, kg/m^2^, mean (SD)	28.7 (5.0)	28.3 (5.0)	29.4 (5.0)	<0.001	27.9 (4.6)	27.6 (4.6)	28.8 (4.6)	<0.001
eGFR, mL/min per 1.73 m^2^, mean (SD)	62.3 (16.4)	62.8 (16.2)	61.6 (16.7)	0.03	80.8 (10.5)	81.0 (10.4)	80.3 (10.7)	<0.001
eGFR categories, *n* (%)								
Stage 1 (≥90)	383 (10.1)	232 (9.6)	151 (10.8)	0.003	3779 (26.5)	2694 (27.0)	1085 (25.4)	0.042
Stage 2 (60–89)	1079 (28.4)	714 (29.7)	365 (26.2)		10,475 (73.5)	7282 (73.0)	3193 (74.6)	
Stage 3a (45–59)	1939 (51.0)	1237 (51.4)	702 (50.3)		Not applicable			
Stage 3b (44–30)	370 (9.7)	204 (8.5)	166 (11.9)					
Stage 4 or higher (<30)	31 (0.8)	20 (0.8)	11 (0.8)					
UACR, mg/mmol, median (IQR)	3.4 (0.9–6.2)	3.4 (0.9–6.1)	3.5 (1.0–6.4)	0.22	0.7 (0.5–1.2)	0.7 (0.4–1.2)	0.7 (0.5–1.2)	0.004
UACR categories, *n* (%)								
No Albuminuria	1427 (37.5)	901 (37.4)	526 (37.7)	0.26	10,336 (72.5)	7207 (72.2)	3129 (73.1)	0.27
Albuminuria	1862 (49.0)	1165 (48.4)	697 (50.0)		Not applicable			
Uncertain	513 (13.5)	341 (14.2)	172 (12.3)		3918 (27.5)	2769 (27.8)	1149 (26.9)	
Nephrotoxic and other meds								
PPIs, *n* (%)	1030 (27.1)	596 (24.8)	434 (31.1)	<0.001	3460 (24.3)	2179 (21.8)	1281 (29.9)	<0.001
NSAIDs, *n* (%)	555 (14.6)	336 (14.0)	219 (15.7)	0.14	2209 (15.5)	1503 (15.1)	706 (16.5)	0.03
Other lipid agents, *n* (%)	205 (5.4)	114 (4.7)	91 (6.5)	0.02	612 (4.3)	340 (3.4)	272 (6.4)	<0.001
Polypharmacy	1385 (36.4)	609 (25.3)	776 (55.6)	<0.001	3422 (24.0)	1657 (16.6)	1765 (41.3)	<0.001
Chronic conditions								
Hypertension and treatments, *n* (%)								
No hypertension	588 (15.5)	454 (18.9)	134 (9.6)	<0.001	4048 (28.4)	3172 (31.8)	876 (20.5)	<0.001
Hypertension on no Rx	678 (17.8)	548 (22.8)	130 (9.3)		3233 (22.7)	2559 (25.7)	674 (15.8)	
Hypertension on Rx (not diuretics and not ACEi/ARB)	321 (8.4)	191 (7.9)	130 (9.3)		910 (6.4)	584 (5.9)	326 (7.6)	
Hypertension on Rx (ACEi/ARB but not diuretics)	1249 (32.9)	715 (29.7)	534 (38.3)		3699 (26.0)	2259 (22.6)	1440 (33.7)	
Hypertension on Rx (diuretics but not ACEi/ARB)	197 (5.2)	217 (5.3)	70 (5.0)		532 (3.7)	334 (3.3)	198 (4.6)	
Hypertension on Rx (ACEi/ARB + diuretics)	769 (20.2)	372 (15.5)	397 (28.5)		1832 (12.9)	1068 (10.7)	764 (17.9)	
Diabetes, *n* (%)								
None	3209 (84.4)	2192 (91.1)	1017 (72.9)	<0.001	12,930 (90.7)	9403 (94.3)	3527 (82.4)	<0.001
Diabetes on no Rx	241 (6.3)	114 (4.7)	127 (9.1)		592 (4.2)	326 (3.3)	266 (6.2)	
Diabetes on Rx (oral hypoglycemic med but not insulin)	291 (7.7)	89 (3.7)	202 (14.5)		650 (4.6)	223 (2.2)	427 (10.0)	
Diabetes on Rx (on insulin)	61 (1.6)	12 (0.5)	49 (3.5)		82 (0.6)	24 (0.2)	58 (1.4)	
Frailty, *n* (%)								
Non‐frail	1921 (50.5)	1221 (50.7)	700 (50.2)	0.37	8802 (61.8)	6208 (62.2)	2594 (60.6)	0.014
Pre‐frail	1762 (46.3)	1104 (45.9)	658 (47.2)		5195 (36.4)	3607 (36.2)	1588 (37.1)	
Frail	119 (3.1)	82 (3.4)	37 (2.7)		257 (1.8)	161 (1.6)	96 (2.2)	
Randomized aspirin	1902 (50.0)	1180 (49.0)	722 (51.8)	0.10	7112 (49.9)	4975 (49.9)	2137 (50.0)	0.93

*Note*: Continuous variables are presented as mean ± SD or median [interquartile range (IQR)] and discrete variables are presented as count (percentage). eGFR was calculated with 2021 Chronic Kidney Disease Epidemiology Collaboration (CKD‐EPI) equation. Albuminuria was defined as urinary albumin to creatinine ratio ≥3 mg/mmol. Other race/ethnicities including Australian Aborigine/Torres Strait Islander, Native American, more than one race, native Hawaiian/Pacific Islander, and those who were not Hispanic and who did not state their ethnicity/race. Diabetes mellitus is defined from self‐report or fasting glucose ≥126 mg/dL or on glucose‐lower medications. Hypertension is defined as blood pressure ≥ 140/90 mmHg or on antihypertensive medications. Polypharmacy was defined as the use of five or more prescription drugs. Other lipid‐lowering medication use included omega‐3‐fatty acid, fenofibrate, ezetimibe, gemfibrozil, nicotinic acid.

Abbreviations: ACEi, angiotensin converting enzyme inhibitor; ARB, angiotensin II receptor blocker; BMI, body mass index; CKD, chronic kidney disease; DBP, diastolic blood pressure; eGFR, estimated glomerular filtration rate; NSAIDs, non‐steroidal anti‐inflammatory drugs; SBP, systolic blood pressure; SD, standard deviation; PPI, proton pump inhibitors; UACR, urine albumin‐creatinine ratio.

**FIGURE 1:**
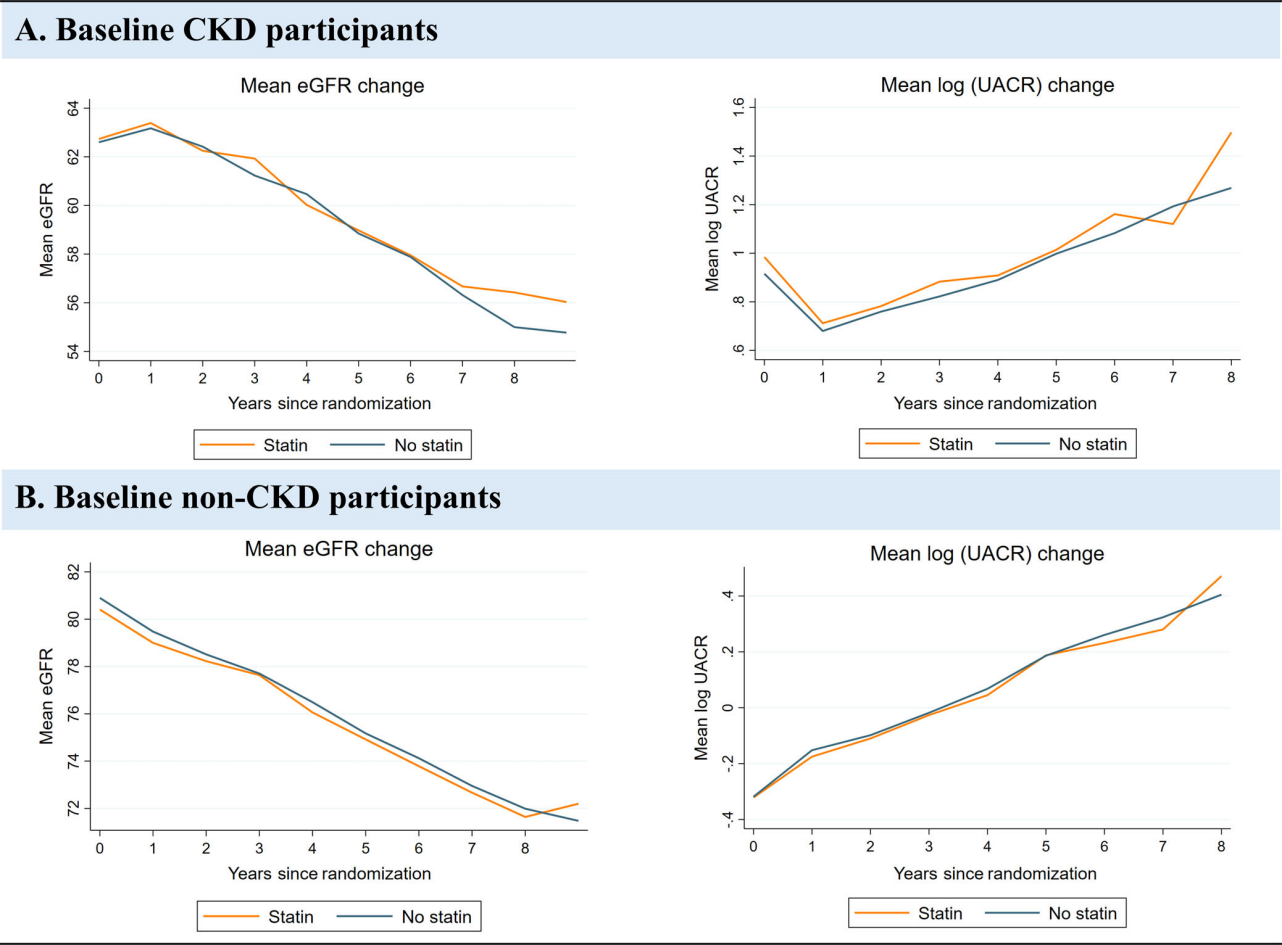
Mean values of estimated glomerular filtration rate and urine albumin‐to‐creatinine ratio at baseline during follow‐up in weighted statin users and nonusers, stratified by baseline chronic kidney disease status. CKD, chronic kidney disease; eGFR, estimated glomerular filtration rate; UACR, urine albumin‐to‐creatinine ratio.

Figure 1 depicts the mean values for eGFR and UACR over time for the IPTW‐weighted participants with and without CKD at baseline. In the linear mixed models, there was no difference in change in eGFR by statin use group. This finding was consistent irrespective of whether participants had CKD at baseline (Table 2). Likewise, when examining change in UACR over time (Table 2), statin use was not associated with change in UACR, and this finding was consistent regardless of baseline CKD status. Finally, when examining incident CKD in participants without CKD at baseline over a median follow‐up of 5.2 years, IPTW‐adjusted Cox proportional‐hazards model revealed there was no association with statin use and incident CKD (HR 0.98, 0.92–1.04; *p* = 0.52) (Table 3). This absence of association was also seen when analyzed via IPTW using a Fine–Gray competing risk model (subdistribution HR 0.98, 0.92–1.05; *p* = 0.60) (Table S2).

**TABLE 2:** The association of statin use versus no use with estimated glomerular filtration rate and urine albumin‐creatinine ratio change among participants with and without chronic kidney disease at baseline, analyzed using inverse‐probability of treatment weighting‐adjusted linear mixed‐effect models.

	eGFR		UACR	
	IPTW‐adjusted coefficient (SE)	*p* value	IPTW‐adjusted coefficient (SE)	*p* value
Baseline CKD participants				
Overall				
Statin	0.146 (0.607)	0.81	0.053 (0.050)	0.29
Visit	−0.843 (0.051)	<0.001	0.033 (0.006)	<0.001
Statin × visit	−0.008 (0.086)	0.92	−0.005 (0.010)	0.62
eGFR ≥ 90 mL/min/1.73 m^2^ (*n* = 344)				
Statin	0.356 (0.521)	0.50	−0.010 (0.090)	0.91
Visit	−1.767 (0.162)	<0.001	−0.091 (0.017)	<0.001
Statin × visit	−0.079 (0.257)	0.76	−0.004 (0.028)	0.89
eGFR 60–89 mL/min/1.73 m^2^ (*n* = 1079)				
Statin	−0.360 (0.673)	0.59	0.098 (0.066)	0.14
Visit	−1.499 (0.101)	<0.001	−0.048 (0.012)	<0.001
Statin × visit	−0.026 (0.183)	0.89	−0.005 (0.019)	0.79
eGFR 45–59 mL/min/1.73 m^2^ (*n* = 1939)				
Statin	−0.143 (0.325)	0.66	0.035 (0.063)	0.58
Visit	−0.388 (0.068)	<0.001	0.097 (0.008)	<0.001
Statin × visit	0.013 (0.109)	0.91	−0.005 (0.013)	0.71
eGFR < 45 mL/min/1.73 m^2^ (*n* = 401)				
Statin	0.543 (0.843)	0.52	0.085 (0.151)	0.57
Visit	−0.396 (0.134)	0.003	0.150 (0.018)	<0.001
Statin × visit	0.203 (0.236)	0.39	−0.032 (0.032)	0.31
Baseline non‐CKD participants				
Overall				
Statin	−0.429 (0.205)	0.036	−0.010 (0.015)	0.51
Visit	−1.104 (0.020)	<0.001	0.092 (0.002)	<0.001
Statin × visit	0.034 (0.037)	0.35	−0.001 (0.004)	0.84
eGFR ≥ 90 mL/min/1.73 m^2^ (*n* = 3779)				
Statin	−0.197 (0.182)	0.28	−0.053 (0.029)	0.07
Visit	−1.545 (0.035)	<0.001	0.083 (0.004)	<0.001
Statin × visit	0.102 (0.065)	0.11	−0.004 (0.007)	0.58
eGFR 60–89 mL/min/1.73 m^2^ (*n* = 10,475)				
Statin	−0.140 (0.201)	0.49	0.006 (0.018)	0.72
Visit	−0.949 (0.025)	<0.001	0.095 (0.003)	<0.001
Statin × visit	0.001 (0.044)	0.99	0.0001 (0.005)	0.99

*Note*: Covariates used for generating inverse‐probability weights included age, sex, country, race, education level, smoking status, alcohol use, mean SBP, mean DBP, mean BMI, hypertension, diabetes, polypharmacy, NSAIDs med use, PPI use, other lipid‐lowering medication use, frailty status, randomized aspirin, baseline eGFR, and UACR levels. The coefficient of statin × visit interaction was interpreted as mean annual difference in eGFR or log UACR between baseline statin users and nonusers. The coefficient of statin main effect was interpreted as the mean baseline difference in eGFR or log UACR between baseline statin users and nonusers.

**TABLE 3:** The association between statin use and incident chronic kidney disease (CKD) events among participants without CKD at baseline, analyzed using inverse‐probability of treatment weighting‐adjusted Cox proportional‐hazards model.

	Statin users, Event/total (IR)	No users, Event/total (IR)	Unadjusted HR (95% CI)	*p* value	IPTW‐adjusted HR (95% CI)	*p* value
Total	1757/4278 (81.1)	3782/9976 (72.5)	1.12 (1.06–1.19)	<0.001	0.98 (0.92–1.04)	0.52
Subgroup						
eGFR ≥90	272/1085 (43.8)	696/2694 (45.5)	0.96 (0.84–1.11)	0.61	0.81 (0.69–0.94)	0.007
eGFR 60–89	1485/3193 (96.1)	3086/7282 (83.7)	1.15 (1.08–1.22)	<0.001	1.01 (0.94–1.08)	0.78

*Note*: Incident CKD (*n* = 5539) was defined as eGFR < 60 mL/min per 1.73 m^2^ or UACR > 3 mg/mmol. The median follow‐up was 5.2 (3.0–6.9) years. Incidence rate (IR) is incidence rate per 1000 person/year in the unweighted population.

Abbreviations: CI, confidence interval; CKD, chronic kidney disease; HR, hazards ratio; IPTW, inverse‐probability treatment weighting.

**FIGURE 2:**
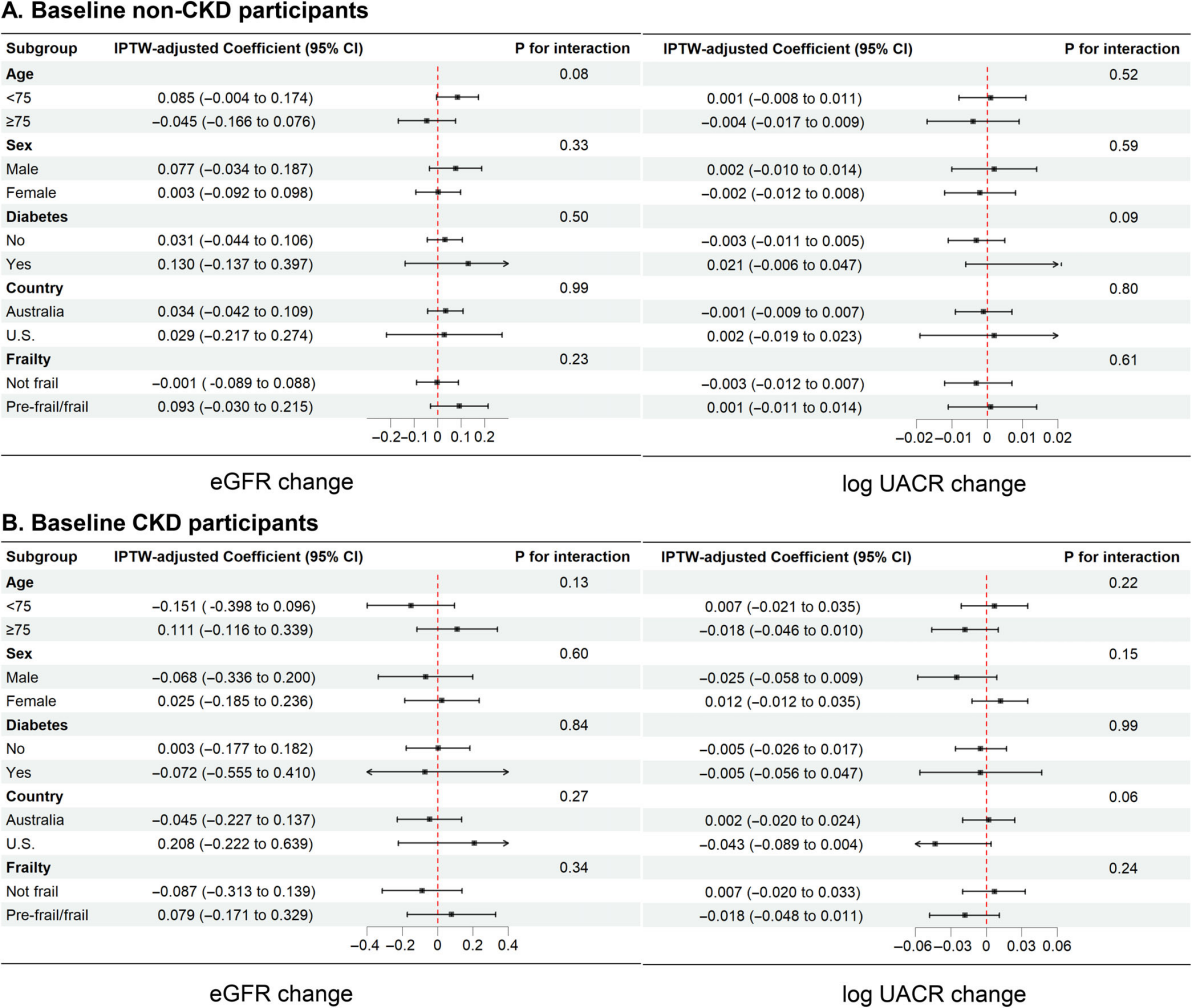
Subgroup analysis for the association of statin use versus no use with estimated glomerular filtration rate and urine albumin‐to‐creatinine ratio change over time among participants with and without chronic kidney disease at baseline. CKD, chronic kidney disease; eGFR, estimated glomerular filtration rate; IPTW, inverse‐probability treatment weighting; SE, standard error; UACR, urine albumin‐to‐creatinine ratio.

Our findings were consistent across several secondary analyses. No significant difference was observed in the association with rosuvastatin use and eGFR change (Table S3) or UACR change (Table S4) in participants with and without CKD at baseline when compared to other statins. Similarly, rosuvastatin use was not associated with incident CKD in participants without CKD at baseline (Table S5) when compared to other individual statin agents. Finally, there was no difference in incident CKD seen with use of rosuvastatin, compared to other statins, in a secondary analysis utilizing the IPTW Fine–Gray competing risk model (Table S6). Subgroup analyses found no significant interactions between statin and age, sex, diabetes, country, and frailty status on any of the study outcomes (Figure 2, Figure S2).

## DISCUSSION

In this analysis of a large older adult cohort from the ASPREE study, we found no evidence of an association between statin use and kidney function. There was no association with statin use and changes in eGFR or UACR over time in participants with and without CKD at baseline and no change in risk of incident CKD in participants without CKD at baseline. Finally, when examined individually, the association between rosuvastatin use and eGFR change, UACR change, and incident CKD was not different compared to the association between these kidney function parameters and use of other individual statins.

Although our study does not lend support to the hypothesis that statins improve kidney outcomes, it does suggest that statins, including rosuvastatin, can be safely used in older adults, both with and without CKD, with regard to kidney health. While older adults are at greatest risk of cardiovascular events and kidney function decline, they are also the population at highest risk of adverse effects from medications; therefore, evidence demonstrating no negative association between statin use and kidney function provides an equally important message to one of kidney benefit. This is a key finding at a critical time as we await results from ongoing, landmark randomized prospective trials (STAREE and PREVENTABLE) which will provide long‐awaited information related to the effects of statins on survival free of dementia and major disability and cardiovascular events when used for primary prevention in older adults.

Our study has several strengths, including a large sample size, long duration of follow‐up, and regular annual laboratory testing of kidney function. Our study also includes limitations. Only 0.2% of participants (*n* = 42) had stage 4 or 5 CKD, therefore our results may not apply to patients with advanced kidney disease. This limitation is particularly important with regard to our analysis of rosuvastatin. Further analysis is needed to make conclusions regarding the safety of rosuvastatin use in older adults with stage 4 or 5 CKD. Our study included mainly White participants, limiting the generalizability to more racially diverse populations. We did not collect data on the length of statin use prior to study entry so it is not possible to conclude if our findings apply specifically to long‐term versus newly initiated statin therapy. Additionally, our study does not assess change in statin therapy through the study period, however, in a previous ASPREE analysis it was demonstrated that a large majority of participants did not change their statin use from baseline (most statin users remained users and nonusers remained nonusers).^19^ Our analysis does not include statin dosing which eliminates our ability to assess for dose‐related positive or negative associations between statins and kidney function. Given that ASPREE participants were required to be free of CVD at study entry, it may be presumed that lower statin doses were more commonly utilized. Therefore, whether our results can be generalized to older adults using high‐dose statins is unclear. The retrospective nature of the trial is a limitation. Indication bias is a significant consideration in this study given the retrospective nature of the analysis, although we attempted to address this by utilizing IPTW.

In summary, we observed that statin use is not associated with changes in kidney function over time in older adults with or without CKD. Furthermore, we did not identify an increased risk of kidney harm associated with use of rosuvastatin compared to other statins. While our findings did not uncover a relationship between statin use and improved kidney health, the data does support the proposal that use of statins in this population is safe, as it relates to kidney health. These findings do not support the use of statins for preservation of kidney function or prevention of kidney disease, but also suggest that providers should not be limited by concerns related to potential kidney harm when deciding to use statins for other indications.

## AUTHOR CONTRIBUTIONS

All authors met the criteria for authorship as follows: study concept and design (Michelle A. Fravel, Michael E. Ernst, Robyn L. Woods, Suzanne G. Orchard, Kevan R. Polkinghorne, Rory Wolfe, James B. Wetmore, Mark R. Nelson, Elisa Bongetti, Anne M. Murray, Sophia Zoungas, Zhen Zhou), acquisition of data (Rory Wolfe, Zhen Zhou), analysis and interpretation of data (Michelle A. Fravel, Michael E. Ernst, Robyn L. Woods, Suzanne G. Orchard, Kevan R. Polkinghorne, Rory Wolfe, James B. Wetmore, Mark R. Nelson, Elisa Bongetti, Anne M. Murray, Sophia Zoungas, Zhen Zhou), and preparation of manuscript (Michelle A. Fravel, Michael E. Ernst, Robyn L. Woods, Suzanne G. Orchard, Kevan R. Polkinghorne, Rory Wolfe, James B. Wetmore, Mark R. Nelson, Elisa Bongetti, Anne M. Murray, Sophia Zoungas, Zhen Zhou). All authors approved the final manuscript. No unnamed contributor played a role in manuscript preparation.

## CONFLICT OF INTEREST STATEMENT

All the authors declare that they have no conflicts of interest with regard to this manuscript.

## SPONSOR'S ROLE

The ASPREE study was supported by the National Institute on Aging (NIA) and the National Cancer Institute at the National Institutes of Health (NIH) (grant numbers U01AG029824, U19AG062682); the National Health and Medical Research Council of Australia (NHMRC) (grant numbers 334047, 1127060); Monash University; and the Victorian Cancer Agency. The sponsors had no role in the design, methods, data collection, analysis, or preparation of the manuscript.

## Supporting information


**Table S1.** Statin use by eGFR categories among participants with and without CKD at baseline.
**Table S2**. The association between statin use and incident CKD events among participants without CKD at baseline, analyzed using IPTW Fine–Gray competing risk model.
**Table S3**. The association of rosuvastatin versus another statin class use with eGFR change over time among participants with and without CKD at baseline, analyzed using IPTW‐adjusted linear mixed‐effect models.
**Table S4**. The association of rosuvastatin versus other statin classes with UACR change over time among participants with and without CKD at baseline, analyzed using IPTW‐adjusted linear mixed‐effect models.
**Table S5**. The association between rosuvastatin use versus other statin class and incident CKD events in participants without CKD at baseline, analyzed using IPTW‐adjusted Cox proportional‐hazards model.
**Table S6**. The association between rosuvastatin use versus other statin class and incident CKD among participants without CKD at baseline, analyzed using IPTW Fine–Gray competing risk model.
**Figure S1**. Participant selection flowchart.
**Figure S2**. Subgroup analysis for the association between statin use and incident CKD events among participants without CKD at baseline.

## References

[1] Ndumele CE , Rangaswami J , Chow SL , et al. Cardiovascular‐kidney‐metabolic health: a presidential advisory from the American Heart Association. Circulation. 2023;148(20):1606‐1635. 37807924 10.1161/CIR.0000000000001184

[2] Ndumele CE , Neeland IJ , Tuttle KR , et al. A synopsis of the evidence for the science and clinical management of cardiovascular‐kidney‐metabolic (CKM) syndrome: a scientific statement from the American Heart Association. Circulation. 2023;148(20):1636‐1664. doi:10.1161/CIR.0000000000001186 37807920

[3] Liao JK , Laufs U . Pleiotropic effects of statins. Annu Rev Pharmacol Toxicol. 2005;45:89‐118. doi:10.1146/annurev.pharmtox.45.120403.095748 15822172 PMC2694580

[4] Choudhary A , Rawat U , Kumar P , Mittal P . Pleotropic effects of statins: the dilemma of wider utilization of statin. Egypt Heart J. 2023;75(1):1. doi:10.1186/s43044-023-00327-8 36602642 PMC9816367

[5] Haynes R , Lewis D , Emberson J , et al. Effects of lowering LDL cholesterol on progression of kidney disease. J Am Soc Nephrol. 2014;25(8):1825‐1833. doi:10.1681/ASN.2013090965 24790178 PMC4116066

[6] Esmeijer K , Dekkers OM , de Fijter JW , Dekker FW , Hoogeveen EK . Effect of different types of statins on kidney function decline and proteinuria: a network meta‐analysis. Sci Rep. 2019;9(1):16632. doi:10.1038/s41598-019-53064-x 31719617 PMC6851118

[7] Zhao L , Li S , Gao Y . Efficacy of statins on renal function in patients with chronic kidney disease: a systematic review and meta‐analysis. Ren Fail. 2021;43(1):718‐728. doi:10.1080/0886022X.2021.1915799 33926359 PMC8901279

[8] You HS , Shin SJ , Kim J , Kang HT . Statin use and incidence of chronic kidney disease in hypercholesterolemia patients with normal renal function. Am J Nephrol. 2021;52(12):940‐948. doi:10.1159/000520532 34864729

[9] Zhao M , Ren L , Zhou Z , Wang T , Li J . The association between statin use and risk of chronic kidney disease in community‐dwelling older people in Shanghai. China Clin Epidemiol. 2022;14:779‐788. doi:10.2147/CLEP.S360395 35782995 PMC9242432

[10] Shin JI , Fine DM , Sang Y , et al. Association of rosuvastatin use with risk of hematuria and proteinuria. J Am Soc Nephrol. 2022;33(9):1767‐1777. doi:10.1681/ASN.2022020135 35853713 PMC9529194

[11] Joseph J , Pajewski NM , Dolor RJ , et al. Pragmatic evaluation of events and benefits of lipid lowering in older adults (PREVENTABLE): trial design and rationale. J Am Geriatr Soc. 2023;71(6):1701‐1713. doi:10.1111/jgs.18312 37082807 PMC10258159

[12] Zoungas S , Curtis A , Spark S , et al. Statins for extension of disability‐free survival and primary prevention of cardiovascular events among older people: protocol for a randomised controlled trial in primary care (STAREE trial). BMJ Open. 2023;13(4):e069915. doi:10.1136/bmjopen-2022-069915 PMC10083753 37012015

[13] McNeil JJ , Nelson MR , Woods RL , et al. Effect of aspirin on all‐cause mortality in the healthy elderly. N Engl J Med. 2018;379(16):1519‐1528. doi:10.1056/NEJMoa1803955 30221595 PMC6433466

[14] McNeil JJ , Wolfe R , Woods RL , et al. Effect of aspirin on cardiovascular events and bleeding in the healthy elderly. N Engl J Med. 2018;379(16):1509‐1518. doi:10.1056/NEJMoa1805819 30221597 PMC6289056

[15] McNeil JJ , Woods RL , Nelson MR , et al. Effect of aspirin on disability‐free survival in the healthy elderly. N Engl J Med. 2018;379(16):1499‐1508. doi:10.1056/NEJMoa1800722 30221596 PMC6426126

[16] Ernst ME , Broder JC , Wolfe R , et al. Health characteristics and aspirin use in participants at the baseline of the ASPirin in reducing events in the elderly ‐ eXTension (ASPREE‐XT) observational study. Contemp Clin Trials. 2023;130:107231. doi:10.1016/j.cct.2023.107231 37196887 PMC10330669

[17] Inker LA , Eneanya ND , Coresh J , et al. New creatinine‐ and cystatin C‐based equations to estimate GFR without race. N Engl J Med. 2021;385(19):1737‐1749. doi:10.1056/NEJMoa2102953 34554658 PMC8822996

[18] Kidney Disease: Improving global outcomes (KDIGO) CKD Work Group . KDIGO 2024 clinical practice guideline for the evaluation and management of chronic kidney disease. Kidney Int. 2024;105(4S):S117‐S314. 38490803 10.1016/j.kint.2023.10.018

[19] Zhou Z , Ofori‐Asenso R , Curtis AJ , et al. Association of statin use with disability‐free survival and cardiovascular disease among healthy older adults. J Am Coll Cardiol. 2020;76(1):17‐27. 32616158 10.1016/j.jacc.2020.05.016 PMC7967891

