# Supplementary material for: Effects of statins on kidney function in older adults
Source: J Am Geriatr Soc. 2024 Dec 18;73(4):1082–93. doi: 10.1111/jgs.19319 (PMC11970218; doi:10.1111/jgs.19319)
Supplement: Supplementary file 1 — Table S1. Statin use by eGFR categories among participants with and without CKD at baseline. Table S2. The association between statin use and incident CKD events among participants without CKD at baseline, analyzed using IPTW Fine–Gray competing risk model. Table S3. The association of rosuvastatin versus another statin class use with eGFR change over time among participants with and without CKD at baseline, analyzed using IPTW‐adjusted linear mixed‐effect models. Table S4. The association of rosuvastatin versus other statin classes with UACR change over time among participants with and without CKD at baseline, analyzed using IPTW‐adjusted linear mixed‐effect models. Table S5. The association between rosuvastatin use versus other statin class and incident CKD events in participants without CKD at baseline, analyzed using IPTW‐adjusted Cox proportional‐hazards model. Table S6. The association between rosuvastatin use versus other statin class and incident CKD among participants without CKD at baseline, analyzed using IPTW Fine–Gray competing risk model. Figure S1. Participant selection flowchart. Figure S2. Subgroup analysis for the association between statin use and incident CKD events among participants without CKD at baseline. [file JGS-73-1082-s001.pdf]

Supplementary Table 1. Statin use by eGFR categories among participants with and without CKD at baseline.

eGFR (ml/min/1.73m^2)	No use	Atorvastatin	Simvastatin	Rosuvastatin	Other statins
**Baseline CKD participants**					
**≥90 (n=344)**	207 (60%)	52 (15%)	39 (11%)	36 (10%)	10 (3%)
**60-89 (n=1079)**	714 (66%)	133 (12%)	119 (11%)	96 (9%)	17 (2%)
**45-59 (n=1939)**	1237 (64%)	264 (14%)	192 (10%)	186 (10%)	60 (3%)
**<45 (n=401)**	224 (56%)	77 (19%)	46 (11%)	36 (9%)	18 (4%)
**Total**	2382	526	396	354	105[a]
**Baseline non-CKD participants**					
**≥90 (n=3,779)**	2694 (71%)	417 (11%)	309 (8%)	287 (8%)	72 (2%)
**60-89 (n=10,475)**	7282 (70%)	1206 (12%)	964 (9%)	816 (8%)	207 (2%)
**Total**	9976	1623	1273	1103	279[b]

[a] Other statins include 82 pravastatin, 5 fluvastatin, 18 lovastatin.

[b] Other statins include 235 pravastatin, 11 fluvastatin, 32 lovastatin, 1 pitavastatin.

Supplementary Table 2. The association between statin use and incident CKD events among participants without CKD at baseline, analysed using IPTW Fine-Gray competing risk model.

	**Unadjusted SHR (95% CI)**	**P value**	**IPTW-adjusted SHR (95% CI)**	**P value**
**Total**	1.12 (1.06-1.19)	<0.001	0.98 (0.92-1.05)	0.60
**Subgroup**				
eGFR ≥90	0.97 (0.84-1.12)	0.67	0.81 (0.70-0.95)	0.009
eGFR 60-89	1.15 (1.08-1.22)	<0.001	1.01 (0.95-1.08)	0.74

Abbreviations: CI, confidence interval; CKD, chronic kidney disease; IPTW, inverse-probability treatment weighting; SHR, sub-distribution hazards ratio.

Supplementary Table 3. The association of rosuvastatin versus another statin class use with eGFR change over time among participants with and without CKD at baseline, analysed using IPTW-adjusted linear mixed-effect models.

	IPTW-adjusted Coefficient (SE)	P value
**Baseline CKD participants**		
Rosuvastatin vs. Atorvastatin		
Rosuvastatin	1.437 (1.103)	0.19
Visit	-0.873 (0.099)	<0.001
Rosuvastatin*visit	0.003 (0.154)	0.99
Rosuvastatin vs. Simvastatin		
Rosuvastatin	0.903 (1.156)	0.43
Visit	-0.719 (0.128)	<0.001
Rosuvastatin*visit	-0.112 (0.187)	0.55
Rosuvastatin vs. Other statins		
Rosuvastatin	3.006 (1.865)	0.11
Visit	-0.584 (0.291)	0.045
Rosuvastatin*visit	-0.191 (0.322)	0.55
**Baseline non-CKD participants**		
Rosuvastatin vs. Atorvastatin		
Rosuvastatin	0.009 (0.101)	0.93
Visit	0.058 (0.012)	<0.001
Rosuvastatin*visit	-0.025 (0.022)	0.25
Rosuvastatin vs. Simvastatin		
Rosuvastatin	0.104 (0.122)	0.39
Visit	0.029 (0.014)	0.045
Rosuvastatin*visit	0.007 (0.026)	0.78
Rosuvastatin vs. other Statins		
Rosuvastatin	0.276 (0.197)	0.16
Visit	0.060 (0.028)	0.035
Rosuvastatin*visit	-0.025 (0.036)	0.48

Abbreviations: CKD, chronic kidney disease; eGFR, estimated glomerular filtration rate; IPTW, inverse-probability treatment weighting; SE, standard error.

Supplementary Table 4. The association of rosuvastatin versus other statin classes with UACR change over time among participants with and without CKD at baseline, analysed using IPTW-adjusted linear mixed-effect models.

	IPTW-adjusted Coefficient (SE)	P value
**Baseline CKD participants**		
Rosuvastatin vs. Atorvastatin		
Rosuvastatin	0.009 (0.101)	0.93
Visit	0.058 (0.012)	<0.001
Rosuvastatin*visit	-0.025 (0.022)	0.25
Rosuvastatin vs. Simvastatin		
Rosuvastatin	0.104 (0.122)	0.39
Visit	0.029 (0.014)	0.045
Rosuvastatin*visit	0.007 (0.026)	0.78
Rosuvastatin vs. Other statins		
Rosuvastatin	0.276 (0.197)	0.16
Visit	0.060 (0.028)	0.035
Rosuvastatin*visit	-0.025 (0.036)	0.48
**Baseline non-CKD participants**		
Rosuvastatin vs. Atorvastatin		
Rosuvastatin	0.010 (0.031)	0.75
Visit	0.095 (0.005)	<0.001
Rosuvastatin*visit	0.000 (0.009)	0.97
Rosuvastatin vs. Simvastatin		
Rosuvastatin	-0.029 (0.035)	0.41
Visit	0.091 (0.006)	<0.001
Rosuvastatin*visit	0.005 (0.009)	0.63
Rosuvastatin vs. other Statins		
Rosuvastatin	-0.064 (0.062)	0.30
Visit	0.110 (0.015)	<0.001
Rosuvastatin*visit	-0.017 (0.017)	0.32

Abbreviations: CKD, chronic kidney disease; IPTW, inverse-probability treatment weighting; SE, standard error; UACR, urine albumin-to-creatinine ratio.

Supplementary Table 5. The association between rosuvastatin use versus other statin class and incident CKD events in participants without CKD at baseline, analysed using IPTW-adjusted Cox proportional-hazards model.

	**Rosuvastatin Event/total (IR)**	**Control Event/total (IR)**	**Unadjusted HR**	**P value**	**IPTW-adjusted HR (95% CI)**	**P value**
Rosuvastatin vs. Atorvastatin	424/1103 (76.8)	668/1623 (81.0)	0.95 (0.84-1.07)	0.37	0.97 (0.86-1.10)	0.61
Rosuvastatin vs. Simvastatin	424/1103 (76.8)	549/1273 (85.2)	0.89 (0.79-1.01)	0.08	0.93 (0.81-1.06)	0.27
Rosuvastatin vs. other statins	424/1103 (76.8)	116/279 (79.5)	0.96 (0.78-1.17)	0.67	1.03 (0.80-1.32)	0.81

Abbreviations: CI, confidence interval; CKD, chronic kidney disease; IPTW, inverse-probability treatment weighting; IR, incidence rate; HR, hazards ratio.

Supplementary Table 6: The association between rosuvastatin use versus other statin class and incident CKD among participants without CKD at baseline, analysed using IPTW Fine-Gray competing risk model.

	**Unadjusted SHR (95% CI)**	**P value**	**IPTW-adjusted HR (95% CI)**	**P value**
Rosuvastatin vs. Atorvastatin	0.94 (0.83-1.06)	0.33	0.96 (0.85-1.09)	0.55
Rosuvastatin vs. Simvastatin	0.89 (0.78-1.01)	0.07	0.93 (0.81-1.06)	0.26
Rosuvastatin vs. Other statins	0.95 (0.77-1.16)	0.61	1.02 (0.80-1.31)	0.86

Abbreviations: CI, confidence interval; CKD, chronic kidney disease; IPTW, inverse-probability treatment weighting; SHR, sub-distribution hazards ratio.

Supplemental Figure 1. Participant selection flow chart.

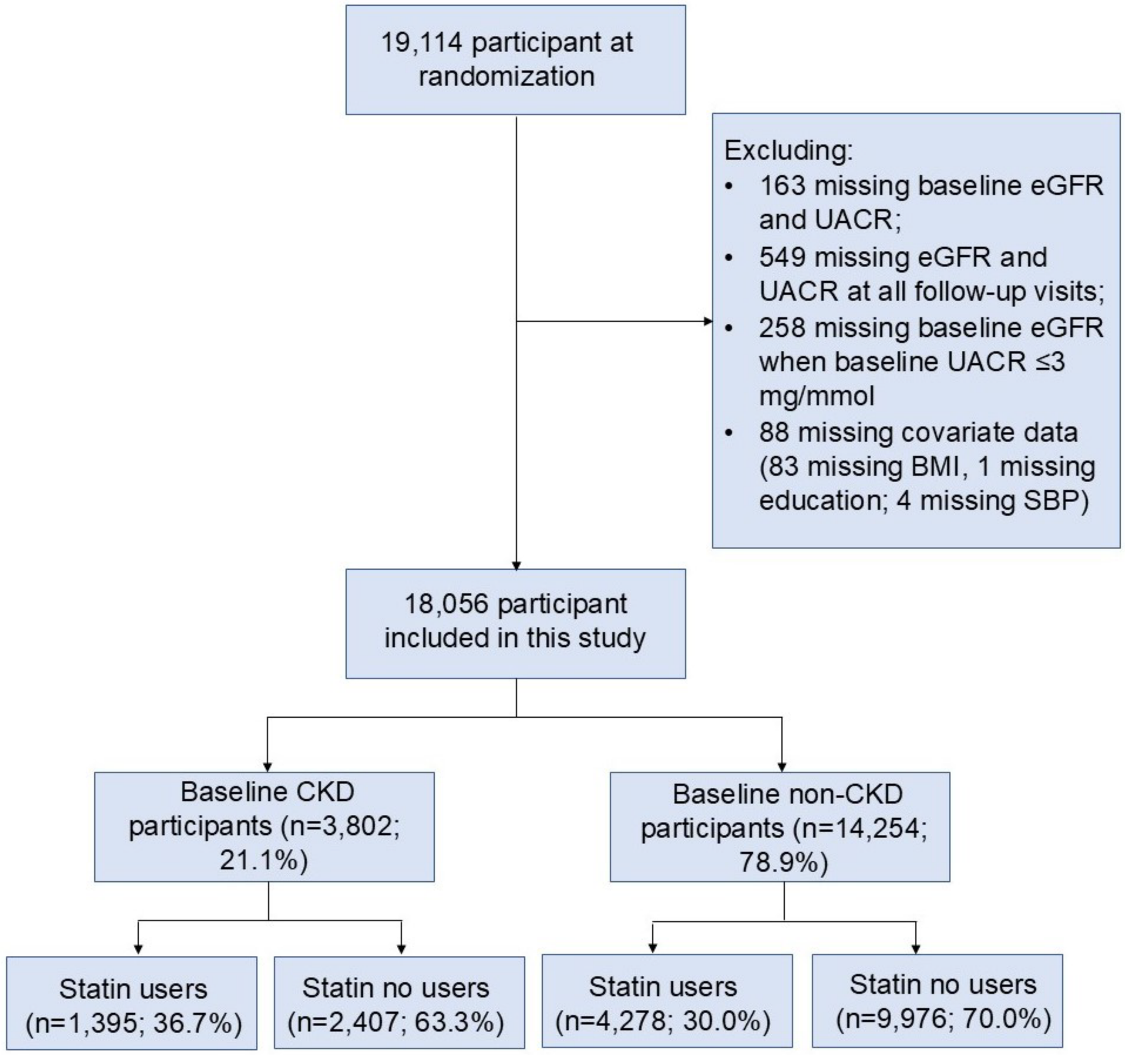

This figure presents a participant selection flow chart. Abbreviations: BMI, body mass index; CKD, chronic kidney disease; eGFR, estimated glomerular filtration rate; SBP, systolic blood pressure; UACR, urine albumin-to-creatinine ratio.

Supplemental Fig 2. Subgroup analysis for the association between statin use and incident CKD events among participants without CKD at baseline.

Subgroup	Event/Total (IR) - Statin	Event/Total (IR) - No Statin	IPTW-adjusted HR (95% CI)	P for interaction
**Age**				0.42
<75	963/2650 (68.9)	2062/6177 (61.6)	0.96 (0.88 to 1.04)	
≥75	794/1628 (103.3)	1720/3799 (92.0)	1.01 (0.92 to 1.10)	
**Sex**				0.30
Male	642/1681 (74.7)	1613/4574 (66.3)	0.94 (0.85 to 1.04)	
Female	1115/2597 (85.3)	2169/5402 (78.0)	1.01 (0.93 to 1.09)	
**Diabetes**				1.00
No	1381/3527 (75.8)	3509/9403 (70.8)	0.98 (0.91 to 1.04)	
Yes	376/751 (109.4)	273/573 (104.0)	0.97 (0.83 to 1.14)	
**Country**				0.83
Australia	1583/3791 (83.3)	3423/8953 (73.8)	0.98 (0.92 to 1.05)	
U.S.	174/487 (65.1)	359/1023 (62.1)	0.96 (0.79 to 1.18)	
**Frailty**				0.07
Not frail	1033/2594 (77.7)	2220/6208 (67.2)	1.03 (0.95 to 1.11)	
Pre-frail/frail	724/1684 (86.4)	1562/3768 (81.6)	0.91 (0.83 to 1.01)	

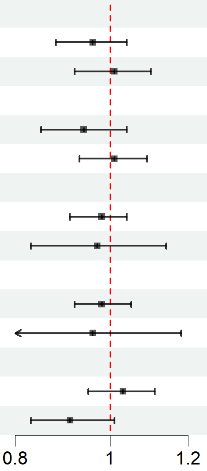

Abbreviations: CI, confidence interval; CKD, chronic kidney disease; IPTW, inverse-probability treatment weighting; HR, hazards ratio.
